# Autoradiographic comparison between [^11^C]PiB and [^18^F]AZD4694 in human brain tissue

**DOI:** 10.1186/s13550-025-01216-8

**Published:** 2025-04-01

**Authors:** Antonio Aliaga, Joseph Therriault, Kely Quispialaya, Arturo Aliaga, Peter Kunach, Arthur C. Macedo, Robert Hopewell, Nesrine Rahmouni, Jean-Paul Soucy, Gassan Massarweh, Marie-Christine Guiot, Tevy Chan, Jesse Klostranec, Aida Mary Abreu Diaz, Andreia Rocha, Giovanna Carello-Collar, Luiza S. Machado, Marco Antônio De Bastiani, Débora Guerini de Souza, Diogo O. Souza, Aline R. Zimmer, Serge Gauthier, Tharick A. Pascoal, Eduardo R. Zimmer, Pedro Rosa-Neto

**Affiliations:** 1 Translational Neuroimaging Laboratory, The McGill University Research Centre for Studies in Aging, Douglas Hospital, McGill University, 6875 La Salle Blvd - FBC Room 3149, Montreal, QC H4H 1R3 Canada; 2 Graduate Program in Biological Sciences: Biochemistry, Universidade Federal Do Rio Grande Do Sul, 2600 Ramiro Barcelos Street, Porto Alegre, Brazil; 3 Research Institute of the McGill University Health Centre, 1001, boul. Decarie -Bloc E, Offices ES2.1602, Montreal, QC 1001H4A 3J1 Canada; 4 Department of Neurology and Neurosurgery, McGill University, Montreal, Canada; 5 Montreal Neurological Institute, Montreal, Canada; 6 Department of Experimental Medicine, McGill University, Montreal, Canada; 7 Department of Pathology, McGill University Health Center, Montreal, Canada; 8 Department of Diagnostic Radiology, McGill University Health Center, Montreal, Canada; 9 Department of Pharmacology and Physiology, University of Montreal, Montreal, Canada; 10 Department of Psychiatry, Pittsburgh University, Pittsburgh, USA; 11 Department of Pharmacology, Universidade Federal Do Rio Grande Do Sul, Porto Alegre, Brazil; 12 Graduate Program in Biological Sciences: Pharmacology and Therapeutics, Universidade Federal Do Rio Grande Do Sul, Porto Alegre, Brazil; 13 Brain Institute of Rio Grande Do Sul, Universidade Federal Do Rio Grande Do Sul, Porto Alegre, Brazil

**Keywords:** Alzheimer’s disease, [^11^C]PiB, [^18^F]AZD4694 ([^18^F]NAV4694), Autoradiography, Amyloid-PET tracer

## Abstract

**Background:**

Amyloid-β imaging through positron emission tomography (PET) has significantly transformed Alzheimer’s disease (AD) research. [^11^C]PiB has been widely used for imaging β-amyloid plaques due to its high affinity and selectivity for amyloid deposits. [^18^F]AZD4694 is a more recently developed amyloid-PET imaging agent, which structurally resembles PiB and has less non-specific binding in the white matter than other ^18^F-labeled compounds. The purpose of this study is to compare the in vitro binding properties of the amyloid-PET radiotracers [^11^C]PiB and [^18^F]AZD4694 in *post-mortem* human brain tissue. Total binding was assessed by autoradiography in prefrontal, inferior parietal, posterior cingulate cortices and hippocampal sections of healthy control (HC) and AD autopsy-confirmed brain tissues. Furthermore, the displacement of [^18^F]AZD4694 by unlabeled PiB was evaluated in the above-mentioned sections of AD brain tissues.

**Results:**

For both radiotracers, we found significant differences (p < 0.0001) between HC and AD tissues binding in the prefrontal cortex ([^11^C]PiB Cohen’s d = 3.424, [^18^F]AZD4694 Cohen’s d = 5.070), inferior parietal cortex ([^11^C]PiB Cohen’s d = 3.156, [^18^F]AZD4694 Cohen’s d = 3.959), posterior cingulate cortex ([^11^C]PiB Cohen’s d = 1.781, [^18^F]AZD4694 Cohen’s d = 3.434), and hippocampus ([^11^C]PiB Cohen’s d = 1.320, [^18^F]AZD4694 Cohen’s d = 3.696). Higher binding was detected for [^18^F]AZD4694 compared to [^11^C]PiB in AD prefrontal, inferior parietal and posterior cingulate cortices, while binding in the hippocampus was comparable for both radioligands. Strong correlations between [^18^]AZD4694 and [^11^C]PiB were found in the prefrontal (R = 0.959, *p* < 0.0001), inferior parietal (R = 0.893, *p* < 0.0001), posterior cingulate (R = 0.838, *p* = 0.0006) cortices and hippocampus (R = 0.750, *p* < 0.0001). Bland–Altman analyses revealed strong agreement between [^11^C]PiB and [^18^F]AZD4694 in the prefrontal, inferior parietal, and posterior cingulate cortices, but lower agreement in the hippocampus. Displacement studies confirmed high binding affinity of PiB in all tissues, indicating that both amyloid-PET agents compete for the same binding sites.

**Conclusions:**

This head-to-head study provides evidence that while [^18^F]AZD4694 and [^11^C]PiB bindings are highly correlated with both tracers competing for the same binding sites, [^18^F]AZD4694 has a slightly higher effect size when comparing between neuropathologically-confirmed AD and HC brain tissues.

**Supplementary Information:**

The online version contains supplementary material available at 10.1186/s13550-025-01216-8.

## Background

The introduction of amyloid imaging through positron emission tomography (PET) [1] has significantly changed Alzheimer's disease (AD) research. Studies using longitudinal amyloid PET imaging in both autosomal-dominant [2] and sporadic [3, 4] AD cases indicate that amyloid pathology begins accumulating many years before the onset of cognitive symptoms. Because of its excellent fidelity for amyloid-β plaques at autopsy [5], amyloid-PET imaging agents have been approved by Food and Drug Administration (FDA) to detect increased amounts of amyloid-β plaques in the brain cortex [6]. Recently, the Alzheimer’s Association published new criteria for the diagnosis and staging of AD, which claim that detection of abnormal levels of amyloid-β plaques by PET imaging is sufficient to establish an AD diagnosis *in* vivo [7]. Furthermore, several recent clinical trials targeting amyloid-β have used amyloid-PET positivity as an enrollment criterion to increase the probability of patients responding to therapy [8–11], with recent trials using quantitative values to determine eligibility for different trials [12]. In turn, appropriate use recommendations for recent anti-amyloid-β monoclonal antibody therapy require the determination of amyloid-positivity using in vivo biomarkers [13, 14]. Moreover, amyloid-PET is often used as an outcome measure to determine target engagement in anti-amyloid clinical trials [15].

The pioneer and benchmark radiotracer for imaging amyloid-β plaques is the ^11^C-labeled Pittsburgh Compound-B ([^11^C]PiB) [1]. [^11^C]PiB has been widely used over the past twenty years due to its high affinity (Kd = 4.7 nM [16]) and selectivity for mature amyloid-β deposits. The second generation of amyloid tracers such as [^18^F]flutemetamol, [^18^F]florbetaben, and [^18^F]florbetapir ([^18^F]AV45) have also been considered promising tools to diagnose AD. Amyloid imaging tracers labeled with fluorine-18, given its relatively long half-life (109.7 min), enable commercial production at central cyclotron sites and distribution to regional PET facilities [17, 18]. In fact, [^18^F]flutemetamol, [^18^F]florbetaben, and [^18^F]florbetapir have been approved by regulatory agencies in multiple countries to detect increased amounts of amyloid-β plaque deposition. However, these ^18^F-labeled radioligands generally present a high non-specific white matter retention and lower cortical binding compared to [^11^C]PiB, which may act as confounding factor for identifying low levels of amyloid deposition [19, 20]. By contrast, [^18^F]AZD4694 (also known as [^18^F]NAV4694, Kd = 2.3 nM) is a more recently developed amyloid-PET imaging agent, which structurally resembles PiB and has less non-specific binding in the white matter than the other ^18^F-labeled compounds [21–24].

While studies have compared [^18^F]AZD4694 with [^11^C]PiB in vivo, to our knowledge, no studies have compared these two imaging agents in AD *post-mortem* brain tissue. Here, we compare the amyloid-PET imaging agents [^18^F]AZD4694 and [^11^C]PiB in an autoradiography head-to-head study.

## Methods

### Radiosynthesis of [^11^C]PiB and [^18^F]AZD4694

The radioligand 2-[4’-([^11^C]methylamino)phenyl]-6-hydroxybenzothiazole ([^11^C]PiB) was synthesized similarly to a previously published methodology [25]. The tracer 2-[2-[^18^F]fluoro-6-(methylamino)-3-pyridinyl]-1-benzofuran-5-ol ([^18^F]AZD4694) was synthesized according to a methodology previously described by our group [23].

### Tissue samples

Frozen brain tissues were obtained from the Douglas-Bell Canada Brain Bank at the Douglas Mental Health University Institute, Montreal, Canada. The *post-mortem* tissues were classified as AD or as healthy control (HC) tissues by the Consortium to Establish a Registry for Alzheimer’s Disease (CERAD). The tissue collection and analysis were approved by the Brain Bank’s and Douglas Institute’s research ethics boards. First, total binding experiments were carried out in total of 88 HC and 88 AD samples. The study comprised the analysis of the prefrontal cortex, inferior parietal cortex, posterior cingulate cortex and hippocampus sections in 11 HC and 11 AD brains, evaluated with [^11^C]PiB and [^18^F]AZD4694. Second, the displacement of [^18^F]AZD4694 by unlabeled PiB was evaluated in the prefrontal, inferior parietal, and posterior cingulate cortices (three samples per brain region for each PiB concentration), and hippocampus (two samples for each PiB concentration) of AD tissues. A total of 88 samples were used for the displacement study.

### Tissue preparation

Flash-frozen tissues blocks were received and placed inside a rotary cryostat (Leica CM3050) until thawed at − 20 °C. Subsequently, the fixed tissues were prepared for embedding within a supportive medium with paraffin. These tissues were then sliced into 20 µm-thick sections, mounted onto microscope slides upon thawing, and stored at − 80 °C.

### Autoradiography imaging

On the experiment day, the frozen samples were thawed, allowed to air-dry and preincubated in NaHEPES buffer as described in our previous study [26]. Prior to autoradiography imaging, the radiotracers were mixed for 10 min with the same buffer. For the first study, the tissues were air-dried and incubated with [^18^F]AZD4694 (2.22 GBq/µmol, 60 Ci/mmol) during 150 min or [^11^C]PiB (11.17 GBq/µmol, 302 Ci/mmol) during 40 min in the incubation buffer. In the second study, the tissues were simultaneously incubated with [^18^F]AZD4694 (1.74 MBq, 47 µCi) and unlabeled PiB at eight different concentrations (0 to 32 nM) for 150 min. Following incubation, the slides were dipped three times in distilled water (4 °C) and dried under a cool-air stream. Finally, the samples were transferred to phosphor imaging plates (Fuji film) for a 20-min exposure. The plates were imaged using an Amersham Typhoon biomolecular imager with a spatial resolution of 50 µm.

### Quantitative image analysis

In the first study, the autoradiography images were analyzed using FIJI/ImageJ software 1.8.0_322 with 2D regions of interest (ROIs) drawn manually in HC and AD tissue samples and in background or non-target areas (outside the tissue). The activity concentration was measured in four equidistant ROIs in each the grey and white matters of the prefrontal, inferior parietal, and posterior cingulate cortices, one ROI in the hippocampus, and four ROIs in the background (Supplementary Fig. 1A). The total binding was calculated as the average activity concentration in each brain region minus the corresponding average background activity concentration. Finally, all results were normalized by the corresponding mean binding values in HC tissues.

The second set of experiments was designed to evaluate the displacement of [^18^F]AZD4694 by PiB using absolute quantification. Autoradiography calibration was performed with eight concentrations of fluorine-18, ranging from 0.001 kBq/mg (0.18 ηCi/mg) to 1.760 kBq/mg (47.58 ηCi/mg) on the same imaging plate. The activity concentrations correlated linearly with the values measured with the FIJI/ImageJ software (R^2^ = 0.9984, *p* < 0.0001). Three equidistant 2D ROIs were drawn manually in the grey matter of the prefrontal, inferior parietal, and posterior cingulate cortices, one ROI in the hippocampus, and three ROIs in background areas (Supplementary Fig. 1B). The total binding in each brain region was calculated as described previously [26]. Data analyses of IC_50_ were performed using the nonlinear regression algorithm of GraphPad Prism 10 software.

### Statistical analysis

The tissue radioactivity concentrations in HC and AD groups were compared using a two-tailed t-test with Welch’s correction using GraphPad Prism 10. A* p* value equal to or less than 0.05 was considered statistically significant. The effect size was assessed using Cohen’s d. Bland–Altman analyses assessed the agreement between measurements of [^11^C]PiB and [^18^F]AZD4694.

## RESULTS

A summary of the demographic characteristics of the subjects is presented in Table 1. The mean age of the participants was 73.3 years (SD = 11.41), and five of them (22.7%) were women. The HC and AD groups did not differ in terms of age (t(14.65) = 0.0912, *p* = 0.929), sex distribution (t(19.60) = 0.488, p = 0.631), and *post-mortem* delay (t(19.34) = 0.576, *p* = 0.571). However, there was a significant difference in brain weight, which was 15.16% lower in the AD group (t(19.78) = 3.754, *p* = 0.001) as compared to the HC group.

**Table 1:** Summary of the demographic characteristics of the healthy control (HC) and Alzheimer’s disease (AD) subjects whose brains were analyzed

Pathologic diagnosis	Gender	Age at death (yrs)	Post-mortem delay (hrs)	Brain weight (g)
HC	M	60	7.25	1350
HC	F	74	11	1365
HC	M	91	6.75	1070
HC	M	69	12	1225
HC	M	51	26.25	1320
HC	M	61	8.75	1337
HC	M	59	17.67	1443
HC	M	88	8	1150
HC	M	85	26.75	1185
HC	F	95	23.75	1160
HC	M	71	76	1250
AD	M	66	8.5	1075
AD	M	67	10.5	1235
AD	F	77	11.25	855
AD	M	76	24	1005
AD	F	74	18	1080
AD	M	73	26.25	1120
AD	M	63	21	1200
AD	F	88	24.5	955
AD	M	79	19.25	910
AD	M	79	24.75	1210
AD	M	67	96	1110

Autoradiography experiments revealed binding of [^11^C]PiB and [^18^F]AZD6496 in HC and AD brain slices, with higher binding in AD tissues for both radioligands. Autoradiographic evaluation of these tracers in the prefrontal cortex, inferior parietal cortex, posterior cingulate cortex and hippocampus of HC (n = 11) and AD (n = 11) brains are displayed in Supplementary Fig. 2.

### Head-to-head comparison of [^11^C]PiB and [^18^F]AZD4694 binding in prefrontal cortex, inferior parietal cortex, posterior cingulate cortex and hippocampus

**Fig. 1:**
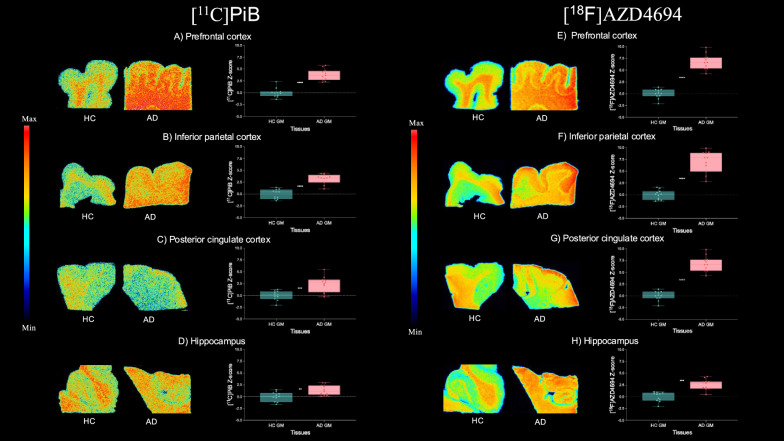
Comparison between [^11^C]PiB and [^18^F]AZD4694 binding in the grey matter of healthy control (HC) and Alzheimer’s disease (AD) brain tissues. Representative autoradiography images and statistical comparison of [^11^C]PiB (n = 11) and [^18^F]AZD4694 (n = 11) binding in the prefrontal cortex (A, E), inferior parietal cortex (B, F), posterior cingulate cortex (C, G), and hippocampus (D, H). Data are expressed as z-scores based on corresponding mean uptake and standard deviation values from HC tissues. Significant differences between HC and AD tissues are present in [^11^C]PiB and [^18^F]AZD4694 binding in prefrontal cortex, inferior parietal cortex, posterior cingulate cortex, and hippocampus grey matter tissues. **p < 0.01, ***p < 0.001, ****p < 0.0001

Significant differences between HC and AD groups were found for both radioligands in the grey matter of the prefrontal cortex ([^11^C]PiB (t-value = 8.030, *p* < 0.0001; Cohen’s d = 3.424), [^18^F]AZD4694 (t-value = 11.89, *p* < 0.0001; Cohen’s d = 5.070), Fig. 1A, E), inferior parietal cortex ([^11^C]PiB (t-value = 7.401, *p* < 0.0001; Cohen’s d = 3.156), [^18^F]AZD4694 (t-value = 9.282, *p* < 0.0001; Cohen’s d = 3.959), Fig. 1B, F), posterior cingulate cortex ([^11^C]PiB (t-value = 4.178, *p* = 0.007; Cohen’s d = 1.781), [^18^F]AZD4694 (t-value = 8.053, *p* < 0.0001; Cohen’s d = 3.434), Fig. 1C, G), and hippocampus ([^11^C]PiB (t-value = 3.096, *p* = 0.006; Cohen’s d = 1.320), [^18^F]AZD4694 (t-value = 5.721, *p* < 0.0001; Cohen’s d = 3.696), Fig. 1D, H). When conducting a subgroup analysis in AD tissues only, we observed significant differences between [^11^C]PiB and [^18^F]AZD4694 total bindings in all regions, as shown in Supplementary Fig. 3.

**Fig. 2:**
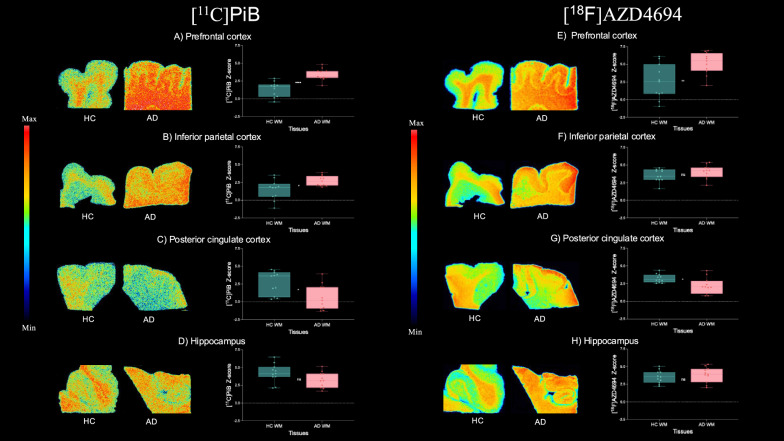
Comparison between [^11^C]PiB and [^18^F]AZD4694 binding in the white matter of healthy control (HC) and Alzheimer’s disease (AD) brain tissues. Representative autoradiography images and statistical comparison of [^11^C]PiB (n = 11) and [^18^F]AZD4694 (n = 11) binding in the prefrontal cortex (A, E), inferior parietal cortex (B, F), posterior cingulate cortex (C, G), and hippocampus (D, H). Data are expressed as z-scores based on corresponding mean uptake and standard deviation values from HC tissues. Significant differences between HC and AD tissues are present in [^11^C]PiB and [^18^F]AZD4694 binding in prefrontal and posterior cingulate cortices white matter tissues. In the inferior parietal cortex, we found significant differences with [^11^C]PiB, but not with [^18^F]AZD4694. No significant differences were found in hippocampus for either radioligand. Not significant (ns), *p < 0.05, **p < 0.01, ****p < 0.0001

The analysis of the white matter also revealed significant differences between HC and AD groups in the prefrontal cortex ([^11^C]PiB (t-value = 5.096, *p* < 0.0001; Cohen’s d = 2.173), [^18^F]AZD4694 (t-value = 3.001, p = 0.008; Cohen’s d = 1.290), Fig. 2A, E), inferior parietal cortex ([^11^C]PiB (t-value = 2.644, *p* = 0.0185; Cohen’s d = 1.127), [^18^F]AZD4694 (t-value = 1.376, p = 0.184; Cohen’s d = 0.586), Fig. 2B, F), posterior cingulate cortex ([^11^C]PiB (t-value = 2.809, *p* = 0.0109; Cohen’s d = 1.198), [^18^F]AZD4694 (t-value = 2.716, *p* = 0.0159; Cohen’s d = 1.158), Fig. 2C, G), and hippocampus [^11^C]PiB (t-value = 1.818, *p* = 0.085; Cohen’s d = 0.775), [^18^F]AZD4694 (t-value = 0.679, *p* = 0.505; Cohen’s d = 0.290), Fig. 2D, H).

#### Correlation between [^11^C]PiB and [.^18^F]AZD4694

**Fig. 3:**
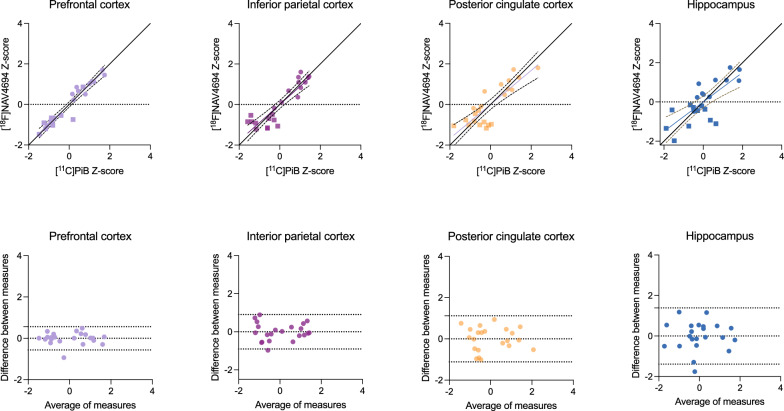
Relationship between [^11^C]PiB and [^18^F]AZD4694 binding. Top: Linear relationships between [^11^C]PiB and [^18^F]AZD4694 in the grey matter of the prefrontal cortex, inferior parietal cortex, posterior cingulate cortex and hippocampus. Circles indicate measurements of tissue from individuals with AD and squares indicate measurements from HC. For the prefrontal, inferior parietal and posterior cingulate cortices, a linear relationship close to the line of origin was observed. Bottom: Bland–Altman analysis assessing bias between [^11^C]PiB and [^18^F]AZD4694 uptakes. Dashed lines indicate limits of agreement. The hippocampus had the largest difference between radioligands

A strong correlation between [^11^C]PiB and [^18^F]AZD4694 binding was confirmed in the prefrontal cortex (R = 0.959, *p* < 0.0001), inferior parietal cortex (R = 0.893, *p* < 0.0001), posterior cingulate cortex (R = 0.838, *p* = 0.0006), and additionally, in the hippocampus (R = 0.750, *p* < 0.0001) (Fig. 3, top). When conducting a subgroup analysis in AD tissue only, we also found a correlation between the radioligands in the prefrontal cortex (R = 0.719, *p* = 0.0002), inferior parietal cortex (R = 0.907, *p* = 0.0009), posterior cingulate cortex (R = 0.627, *p* = 0.0004), and hippocampus (R = 0.638, *p* = 0.003). Bland–Altman analyses revealed strong agreement between [^11^C]PiB and [^18^F]AZD4694 in the prefrontal, inferior parietal, and posterior cingulate cortices, with lower agreement in the hippocampus (Fig. 3, bottom).

### Displacement of [^18^F]AZD4694 by unlabeled PiB

**Fig. 4:**
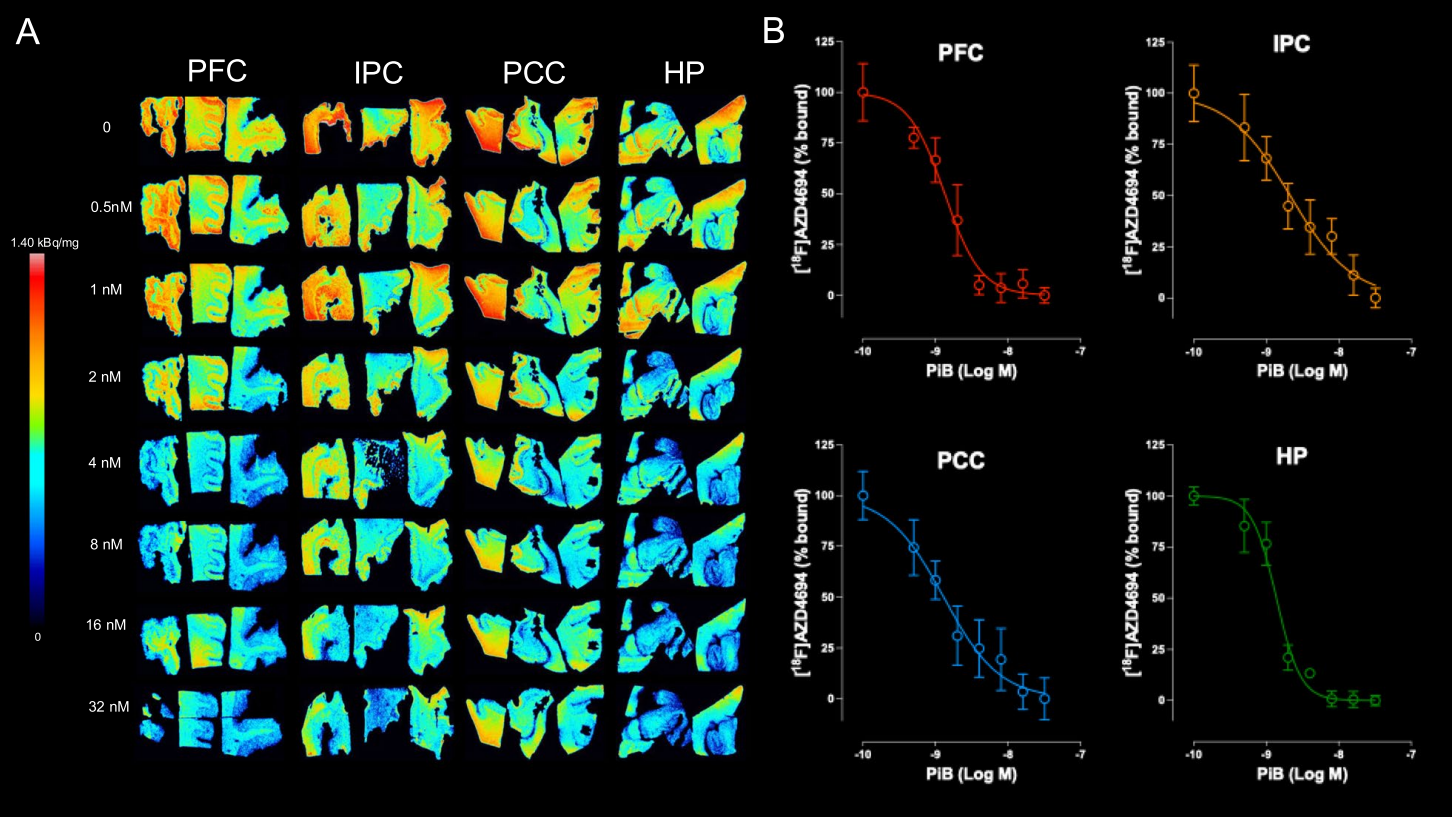
Displacement of [^18^F]AZD4694 by PiB in Alzheimer’s disease (AD) tissues. **A** Autoradiography images of [^18^F]AZD4694 binding following coincubation with PiB (0 to 32 nM) in the prefrontal cortex (PFC), inferior parietal cortex (IPC), posterior cingulate cortex (PCC) and hippocampus (HP) of AD brains. All tissues illustrate that both ligands compete for the same binding sites. **B** Displacement curves of [^18^F]AZD4694 by PiB in PFC, IPC, PCC and HP. Total binding was measured in three equidistant regions of interest (ROIs) placed in the grey matter of the PFC, IPC, and PCC, and one ROI in the HP. Data are expressed as a percentage of total binding in the absence of PiB and represent the mean ± standard deviation in the PFC (n = 3), IPC (n = 3), PCC (n = 3), and HP (n = 2) at each concentration. PiB exhibited a high binding affinity in the PFC (IC_50_ = 1.34 nM, R^2^ = 0.94, slope = − 1.73), IPC (IC_50_ = 2.13 nM, R^2^ = 0.90, slope = −1.01), PCC (IC_50_ = 1.29 nM, R^2^ = 0.90, slope = -1.12) and HP (IC_50_ = 1.38 nM, R^2^ = 0.97, slope = −2.60)

The autoradiography images showing the displacement of [^18^F]AZD4694 by unlabeled PiB from the amyloid-β expressed in AD brain regions, such as the prefrontal cortex, inferior parietal cortex, posterior cingulate cortex, and hippocampus, are presented in Fig. 4A. PiB exhibited a high binding affinity in the prefrontal cortex (IC_50_ = 1.34 nM), inferior parietal cortex (IC_50_ = 2.13 nM), posterior cingulate cortex (IC_50_ = 1.29 nM) and hippocampus (IC_50_ = 1.38 nM) (Fig. 4B).

## DISCUSSION

This head-to-head study evaluated the binding properties of [^11^C]PiB and [^18^F]AZD4694 in *post-mortem* human brain tissue. We report that [^18^F]AZD4694 had a slightly higher affinity for amyloid-β plaques than [^11^C]PiB. Correspondingly, [^18^F]AZD4694 also had a higher effect size when contrasting between neuropathologically-confirmed AD and HC brain tissues. In addition, [^18^F]AZD4694 had a slightly lower binding to the white matter as compared to [^11^C]PiB. Displacement studies revealed that both imaging agents compete for the same binding sites. Taken together, these results support the use of [^18^F]AZD4694 as an excellent imaging agent for in vivo imaging and quantification of amyloid-β plaques.

We observed that in grey matter, [^18^F]AZD4694 had a higher effect size than [^11^C]PiB for distinguishing between AD and HC brain tissues. Specifically, binding in the prefrontal, inferior parietal and posterior cingulate cortices, all brain regions known to accumulate high levels of amyloid-β in AD [27–30], was higher with [^18^F]AZD4694. In these regions, the mean fold-change from HC to AD tissue for this tracer was approximately 7.5x, while for [^11^C]PiB the fold-change was approximately 3x. Furthermore, binding in the hippocampus, known to accumulate amyloid-β plaques relatively later in the natural history of AD [30], was also higher with [^18^F]AZD4694, with a fold-change of approximately 2.5x, and only 1 × for [^11^C]PiB. These results corroborate previous studies suggesting that [^18^F]AZD4694 has a higher effect size for distinguishing between HC and AD, likely due to [^18^F]AZD4694’s higher counting rate during the scanning window by virtue of fluorine-18 longer half-life (109.7 min) compared to carbon-11 (20.4 min), resulting in better images [31]. Additionally, the slightly higher affinity for human amyloid-β fibrils reported for [^3^H]AZD4694 (Kd = 2.3 nM [21]) compared to [^3^H]PiB (Kd = 4.7 nM [16]) may contribute to the higher effect size observed for [^18^F]AZD4694.

In direct head-to-head comparisons of [^18^F]AZD4694 and [^11^C]PiB, we observed strong correlations between regional binding of both radioligands. Both amyloid-PET imaging agents were most closely correlated in the prefrontal cortex and inferior parietal cortex, followed by the posterior cingulate cortex and hippocampus. The prefrontal and inferior parietal cortices also both had the lowest standard deviations between radiotracers. Interestingly, the largest standard deviations between radioligands were observed in the hippocampus, a region where amyloid-PET levels are known to rise in later disease. Even in the hippocampus, however, almost all datapoints were within the limits of agreement, with only one falling beyond of the lower limit of agreement.

In line with the head-to-head comparison findings, displacement studies revealed that both amyloid-PET imaging agents compete for the same binding sites. The IC_50_ values obtained for PiB in prefrontal cortex, inferior parietal cortex, posterior cingulate cortex and hippocampus show a high binding affinity of the ligand. However, the curve for the hippocampus is steeper than a standard curve which might reflect particular affinity differences between [^18^F]AZD4694 and PiB due to the predominance of non-cored plaques in the hippocampus [32, 33]. This result matches the lowest agreement between radiotracers found in Bland–Altman analysis for this tissue. The standard slope observed for prefrontal, inferior parietal, and posterior cingulate cortices demonstrates a better correlation in binding behaviour of [^18^F]AZD4694 across those regions as compared to [^11^C]PiB in same regions. However, it is also possible that the small number of samples used for image analysis contributed to this finding.

[^11^C]PiB has served as the benchmark for head-to-head comparisons of amyloid-PET imaging agents largely because of its favorable binding properties [34, 35] and because of evidence of in vitro binding with cyano-PiB [33]. In fact, for conversion to Centiloids, all imaging agents were first compared to [^11^C]PiB in head-to-head studies [36]. These previous studies have reported that [^11^C]PiB has a higher effect size for differentiating between HC and AD tissues than [^18^F]florbetapir [37], [^18^F]flutemetamol [38], and [^18^F]florbetaben [39]. The only advantage of these other radioligands over [^11^C]PiB is that because they are labeled with fluorine-18, their radioactive half-life of 109.7 min allows for centralized production with the possibility for extensive clinical use [23]. However, [^18^F]AZD4694, which is also labeled with fluorine-18, has been shown to have a higher effect size than [^11^C]PiB, and by extension, other fluorinated amyloid-PET imaging agents. Better effect sizes may translate to superior identification of amyloid-β pathology using PET visual assessments [40], as well as being better suited to capture longitudinal changes in amyloid-PET load [41]. Results from [^18^F]AZD4694 (a.k.a. [^18^F]NAV4694) Phase 2 and 3 studies might reveal the clinical potential of this amyloid imaging agent [42, 43].

The recent approval of the anti-amyloid-β monoclonal antibodies aducanumab, lecanemab, and donanemab in addition to the FDA’s decision to accept amyloid-β reduction as a surrogate marker of clinical efficacy [44], highlights the need for precise measurement of amyloid-β pathology at the individual level [45, 46]. Appropriate use criteria for aducanumab and lecanemab require the presence of abnormal amyloid-β biomarkers prior to initiating therapy [14]. Likewise, most modern ongoing clinical trials for AD require biomarker evidence of amyloid-β plaques for trial eligibility. Furthermore, the several additional monoclonal antibodies currently under evaluation in AD [8, 47] support the expectation that molecular brain imaging in neurodegenerative disease may become a routine clinical tool in the coming years [48].

Our study has limitations which must be mentioned. First, the binding properties described using autoradiography in this study do not perfectly recapitulate in vivo amyloid-PET binding properties. For example, in vivo PET signal using simplified semi-quantitative methods (i.e. the standardized uptake value ratio, SUVR) is influenced by factors such as tracer delivery (cerebral blood flow) and washout [49]. Second, total binding was used instead of specific binding for head-to-head comparison. Determination of specific binding requires measurements of nonspecific binding by incubating the tissues with the tracer and a competitor under steady-state conditions, which are difficult to achieve with [^11^C]PiB due to the fast decay of carbon-11. [^11^C]PiB is the gold standard for evaluating new amyloid radioligands for PET, however, some challenges and potential errors could arise from the autoradiographic comparison between ^18^F-fluorinated and ^11^C-labeled tracers due to differences in the nuclear properties of the radioisotopes. The shorter half-life of carbon-11 (20.4 min) compared to fluorine-18 (109.7 min) possesses challenges to the experiment execution time and molar activity of the radioligand. This limitation was overcome in our study by running a faster incubation and using a higher molar activity for [^11^C]PiB in contrast to [^18^F]AZD4694. The short half-life of carbon-11 also leads to lower counting rates during image acquisition, while the activity decreases, affecting resolution. Moreover, signal-to-noise ratio (SNR) is more favorable for ^18^F-labeled radioligands due to the shorter positron range in tissues for fluorine-18 (2.4 mm) versus carbon-11 (4.2 mm). Although the SNR issues can be mitigated by using larger ROIs, future research is necessary to address this topic in autoradiography imaging. The low number of brain regions sampled is another limitation. However, the regions sampled in this study reflect known areas of amyloid-PET accumulation. Furthermore, while this study focused on contrasting HC from AD, future studies will benefit from looking into the discriminative capacity of different amyloid-PET imaging agents to detect early levels of amyloid-β accumulation, particularly in asymptomatic individuals.

## Conclusion

This head-to-head study provides evidence that [^18^F]AZD4694 and [^11^C]PiB bindings are highly correlated, with these tracers competing for the same binding sites. However, [^18^F]AZD4694 has a slightly higher effect size for discriminating between neuropathologically-confirmed AD and HC brain tissues.

## Supplementary Information

Supplementary file 1.

## Data Availability

The raw data supporting the conclusions of this article will be made available by the authors, without undue reservation.
