# Supplementary material for: Autoradiographic comparison between [11C]PiB and [18F]AZD4694 in human brain tissue
Source: EJNMMI Res. 2025 Apr 1;15:30. doi: 10.1186/s13550-025-01216-8 (PMC11961831; doi:10.1186/s13550-025-01216-8)
Supplement: Supplementary file 1 [file 13550_2025_1216_MOESM1_ESM.pdf]

European Journal of Nuclear Medicine and Molecular Imaging Research

Supplementary Figures

Autoradiographic comparison between [^{11}C]PiB and [^{18}F]AZD4694 in human brain tissue

Antonio Aliaga MSc[1,2,3*], Joseph Therriault PhD[1,4,5*], Kely Quispialaya MD[1,3,5,6], Arturo Aliaga BSc[1,5], Peter Kunach MSc[1,4,5], Arthur C. Macedo MD[1,4,5], Robert Hopewell MSc[5], Nesrine Rahmouni MSc[1,4,5], Jean-Paul Soucy MD MSc[5], Gassan Massarweh PhD[5], Marie-Christine Guiot MD[4,7], Tevy Chan MD[1,4,5], Jesse Klostranec MD, PhD[8], Aida Mary Abreu Diaz PhD[9], Andreia Rocha PhD[10], Giovanna Carello-Collar MSc[2], Luiza S. Machado MSc[2], Marco Antônio De Bastiani PhD[2], Débora Guerini de Souza PhD[2], Diogo O. Souza MD, PhD[2], Aline R. Zimmer PhD[11], Serge Gauthier MD[1,4], Tharick A. Pascoal MD, PhD[10], Eduardo R. Zimmer PhD[1,2,12,13], and Pedro Rosa-Neto MD, PhD[1,3,4,5,6]

[1]Translational Neuroimaging Laboratory, The McGill University Research Centre for Studies in Aging, Douglas Hospital, McGill University, Montreal, Canada.
[2]Graduate Program in Biological Sciences: Biochemistry, Universidade Federal do Rio Grande do Sul, Porto Alegre, Brazil
[3]Research Institute of the McGill University Health Centre, Montreal, Canada.
[4]Department of Neurology and Neurosurgery, McGill University, Montreal, Canada.
[5]Montreal Neurological Institute, Montreal, Canada.
[6]Department of Experimental Medicine, McGill University, Montreal, Canada
[7]Department of Pathology, McGill University Health Center, Montreal, Canada
[8]Department of Diagnostic Radiology McGill University Health Center, Montreal, Canada
[9]Department of Pharmacology and Physiology, University of Montreal, Montreal, Canada
[10]Department of Psychiatry, Pittsburgh University, Pittsburgh, United States
[11]Department of Pharmacology, Universidade Federal do Rio Grande do Sul, Porto Alegre, Brazil
[12]Graduate Program in Biological Sciences: Pharmacology and Therapeutics, Universidade Federal do Rio Grande do Sul, Porto Alegre, Brazil
[13]Brain Institute of Rio Grande do Sul, Universidade Federal do Rio Grande do Sul, Porto Alegre, Brazil

[*] These authors contributed equally.

Corresponding authors:
Pedro Rosa-Neto, MD, PhD. Email: pedro.rosa@mcgill.ca
Eduardo R. Zimmer, PhD. Email: eduardo.zimmer@ufrgs.br

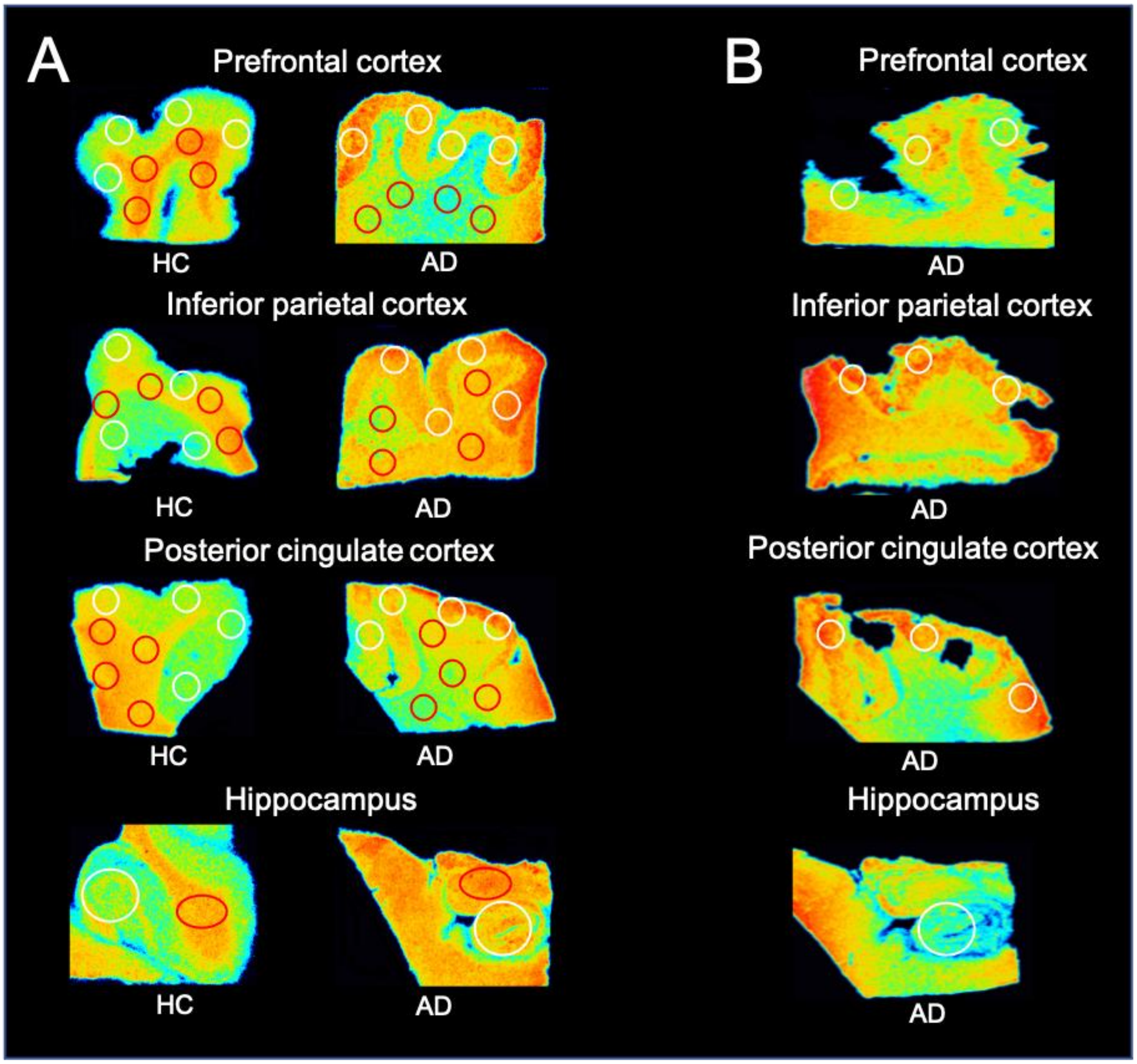

Supplementary Figure 1. Regions of interest used in this study. A) Autoradiography images of the prefrontal, inferior parietal and posterior cingulate cortices were measured in four equidistant regions of interest (ROIs) placed in each the grey matter (GM, white circles) and white matter (WM, red circles). For the hippocampus, one ROI was placed in each the GM and WM. B) Displacement images of the AD tissues were measured in three equidistant ROIs placed in each the prefrontal cortex, inferior parietal cortex, and posterior cingulate cortex GMs, as well as one ROI in the hippocampus. In both, study A and B, the total binding was calculated as the average activity concentration in each brain region minus the corresponding average background (nontarget area outside the tissues) activity concentration.

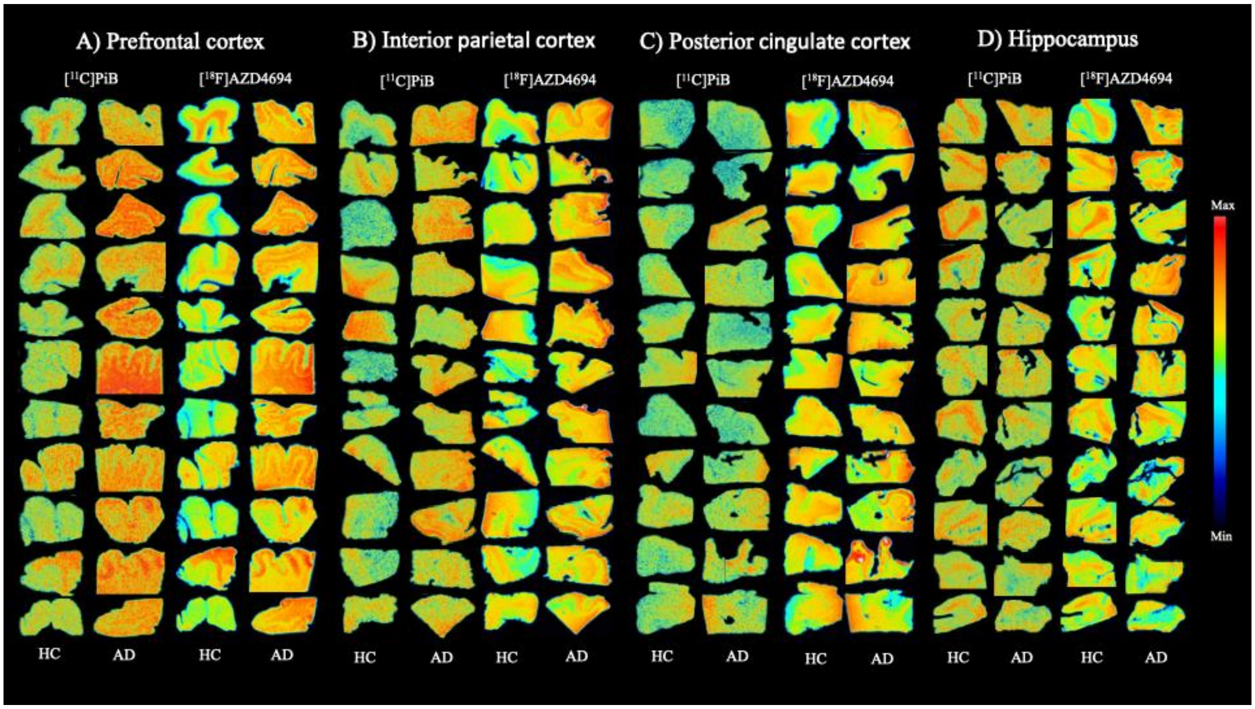

Supplementary Figure 2. Autoradiography images with [^{11}C]PiB and [^{18}F]AZD6494 in healthy control (HC) and Alzheimer's disease (AD) brain tissues. [^{11}C]PiB and [^{18}F]AZD6494 binding was measured in the (A) prefrontal cortex, (B) inferior parietal cortex, (C) posterior cingulate cortex and (D) hippocampus of HC and AD brains.

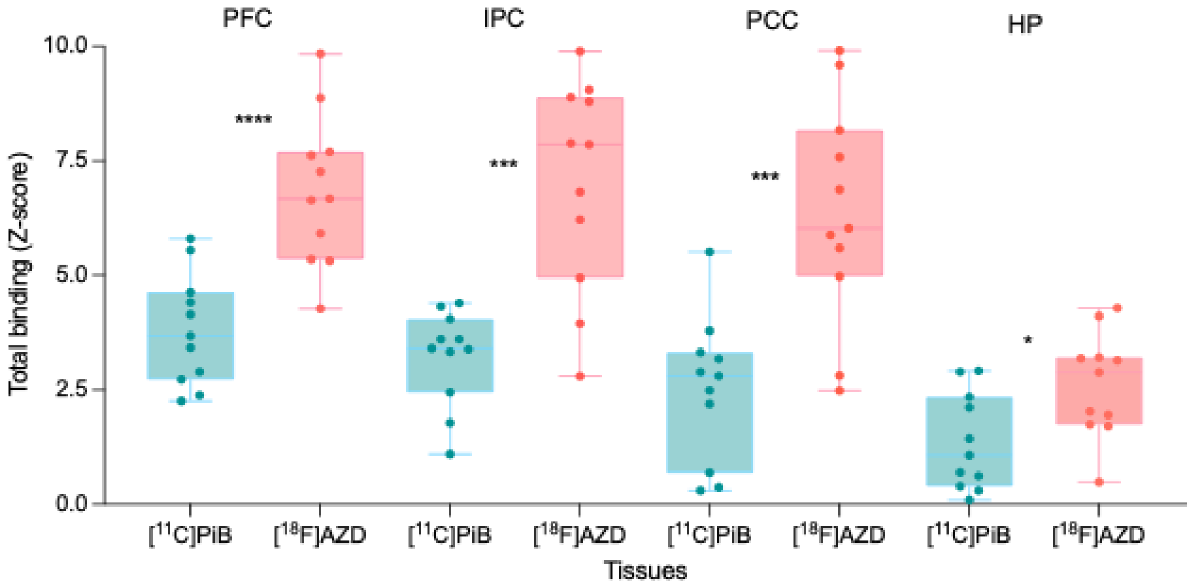

Supplementary Figure 3. Regional uptake of [^{11}C]PiB and [^{18}F]AZD4694 in Alzheimer's disease (AD) brain tissues. Total binding in the grey matter region of the prefrontal cortex (PFC), inferior parietal cortex, (IPC) posterior cingulate cortex (PCC) and hippocampus (HP) of AD tissues. We observed significant differences between [^{11}C]PiB and [^{18}F]AZD4694 ([^{18}F]AZD) in the prefrontal cortex (t-value=4.973, $p<0.0001$), inferior parietal cortex (t-value=4.984, $p=0.0002$), posterior cingulate cortex (t-value=4.425, $p=0.0004$) and hippocampus (t-value=2.694, $p=0.014$). *$p<0.05$, ***$p<0.001$, ****$p<0.0001$.
